# The Effect of Malignant Disease on the Erythrocyte Sedimentation Rate

**DOI:** 10.1038/bjc.1962.6

**Published:** 1962-03

**Authors:** M. A. Peyman


### THE EFFECT OF MALIGNANT DISEASE ON THE ERYTHROCYTE SEDIMENTATION RATE

M. A. PEYMAN

*From Charing Cross Hospital, London, W.C.2*

Received for publication February 12, 1962

SINCE the early observations of Fahraeus (1921) on the suspension-stability of the blood, it has been recognised that malignant disease may often be associated with rapid sedimentation of the erythrocytes. Indeed, Walton (1933) suggested that an increased erythrocyte sedimentation rate (E.S.R.) occurred in all cases of malignant neoplasms with the exception of those arising in certain sites such as the tongue, and Reichel (1936) maintained that the presence of a normal E.S.R. in a doubtful case of malignancy excluded the diagnosis with considerable certainty. More recently, Lipschutz (1953) reported that the E.S.R. was elevated in most patients with malignant disease involving the gastro-intestinal tract, and he concluded that this test was not always accorded the importance it deserved in the differentiation of benign from malignant conditions. However, other workers (Gram, 1929 ; Gregg and Allen, 1939 ; Agnor, 1940 ; Strasser and Weiser, 1954 ; Fearnley, 1957) have claimed that the sedimentation rate is frequently unaffected in cancer especially during the early stages, and Bannick, Gregg and Guernsey (1937) emphasized that normal values are sometimes found even in the presence of widespread metastases.

There has in fact been much speculation not only in regard to the diagnostic value of the E.S.R. in malignant disease but also with respect to the mechanism of rapid sedimentation in these cases. Thus Fahraeus (1921), in his initial studies, stated that " it is difficult to decide whether the pathological generation of tissue itself produces reduction of the suspension-stability of blood or whether this change is only a consequence of tissue destruction or infection ". Certainly, Kourilsky, Decroix and Duwoos (1952) were convinced that the mere growth of cancer tissue in the body was often responsible for an increased E.S.R. On the other hand, Vickers and Duryee (1932) contended that the development of rapid sedimentation in malignancy was usually the result of complications such as secondary infection, while other authors (Cutler, 1932 ; Britton, 1936 ; Bouton, 1938 ; Johnson, 1939 ; Nichols, 1945) have maintained that the degree of acceleration of the E.S.R. in these cases depends upon the amount of tissue destruction.

The present paper describes the results of a clinico-pathological study designed to re-appraise the effect of malignant disease on the E.S.R. Particular attention was paid to the significance of factors such as the site of the primary neoplasm as well as the presence of fever, leucocytosis, secondary infection, anaemia and loss of weight. In addition, the effect of metastases in various sites of the body was studied, and the possible association between the E.S.R. and the morphology of neoplasms, as determined by histological examination, was also investigated.

### MATERIAL, METHODS AND DEFINITION OF FACTORS

The behaviour of the E.S.R. was studied in 300 patients with malignant neoplasms admitted to Charing Cross Hospital between 1956 and 1959. Patients were not included in the series if they showed evidence of disorders which, regardless of the presence of malignant disease, are known to influence the sedimentation rate. However, the case records of 25 patients with multiple myelomatosis were reviewed so that the effect of this disorder on the E.S.R. could be compared and contrasted with that observed in malignant growths.

The presence of malignant disease was always confirmed by histological examination of the primary neoplasm. Tissue specimens were obtained at operation in 125 cases, at autopsy in 110 cases, and at biopsy in 65 subjects.

*Measurement of the E.S.R.*—The E.S.R. was determined by the Westergren method. Consideration was confined in the present study to the values obtained at the end of the first hour. No correction was made for anaemia. In this connection, Alston (1946) maintained that as the E.S.R. was probably influenced by a number of factors, it was meaningless to correct solely for a reduced blood count. The use of correction curves in anaemia has also been criticised by other workers (Bouton, 1938 ; Cutler, Park and Herr, 1938 ; Poole and Summers, 1952 ; Fearnley, 1957).

The E.S.R. of every patient included in this series was measured on at least 3 separate days (not consecutive) during a period extending up to 6 weeks before the removal of tissue specimens for pathological examination. The final E.S.R. was always determined within 14 days of biopsy, operation or post-mortem. For the purpose of the present investigation, the E.S.R. of an individual patient was taken to be the average (i.e. mean) value of the serial observations recorded during the 6-week period. Cases in which the E.S.R. varied by more than 10 mm. in one hour during the period of observation were not accepted for analysis. None of the subjects received radiotherapy or cytotoxic drugs during the period of the E.S.R. determinations.

The normal range of the E.S.R., as determined by the method used in this study (i.e. Westergren technique), was established in 100 subjects in apparent good health. The following results were obtained.

TABLE I.—*Range and Mean Values of E.S.R. in* 100 *Control Subjects*

| Subjects | Mean value mm. in one hour | Standard error | Range mm. in one hour |
|---|---|---|---|
| 50 men (all age groups) | 5·7 | 0·24 | 1–10 |
| 50 women (all age groups) | 6·3 | 0·30 | 1–15 |
| 18–49 age group (25 men and 25 women) | 5·9 | 0·22 | 1–10 |
| 50–84 age group (25 men and 25 women) | 6·1 | 0·31 | 1–15 |

There was no significant difference either between the male and female mean values ($t = 1{\cdot}562 : P = >0{\cdot}05$) or between the mean values of the two age groups ($t = 0{\cdot}534 : P = >0{\cdot}05$). The E.S.R. was never greater than 10 mm. in one hour in the control subjects with the exception of 3 females in the older age group. It was, therefore, decided to accept a range of 1–10 mm. in one hour as the norm for all patients, irrespective of age and sex, included in the present study. (The series of patients with malignant disease consisted of 182 males and 118 females ;

225 patients were over 50 years of age). Sedimentation rates above 10 mm. in one hour were divided on an arbitrary basis into four categories as shown under " Results ".

*Blood count estimations.*—For every patient included in this series, the results of at least two blood counts, performed within 4 days of an E.S.R. determination, were available for analysis. Each blood examination consisted of estimation of the haemoglobin value and a leucocyte count as well as usually a red cell count. The mean of these values was taken to represent the blood count of an individual patient. Anaemia was defined in males as consisting of a mean haemoglobin value of less than 14 g. per cent, and/or a red cell count of less than 4,500,000 per c.mm., and in females as a mean haemoglobin value of less than 12 g. per cent, and/or a red cell count of less than 4,000,000 per c.mm. (Whitby and Britton, 1957). Leucocytosis was defined as a mean white cell count greater than 11,000 per c.m.m. (Whitby and Britton, 1957).

*Analysis of temperature charts.*—During the period of observation, the temperature of every patient was recorded at least once every 12 hours ; if fever was present, additional reports were made at 4-hourly intervals. All temperature readings accepted for inclusion in this study were obtained during the 24 hours before and after an E.S.R. determination (i.e. 48-hour period). The mean of these values was accepted as representing the temperature of an individual patient. Fever was defined as a mean temperature above 99° F. (Oral).

*Evidence of loss of weight.*—Patients were divided into those with and without loss of weight according to the history obtained at the time of admission to hospital. Cases in which doubt existed as to the development of this symptom were not included in the series.

*Evidence of secondary infection.*—Statistical analysis designed to determine the association between the E.S.R. and secondary infection was confined to the group of patients with carcinoma of the bronchus as in those with other neoplasms it was sometimes difficult to confirm the presence of this complication. Unequivocal evidence of secondary infection was obtained in 51 of the 101 patients in the bronchial group (e.g. pneumonia, empyema, lung abscess, infected atelectasis, pneumonia and septic bronchiectasis, etc.). As the E.S.R. of every patient included in this series always represented the mean value of serial observations made over a period extending up to 6 weeks, care was taken to exclude as far as possible from the above group those cases in which secondary infection appeared to be a terminal event, e.g. broncho-pneumonia developing within a few days of death.

*Evidence of systemic metastases.*—Special attention was paid to the effect on the E.S.R. of metastases situated in the following sites : (1) Liver, (2) Skeletal system, (3) Lungs, (4) Brain. Evidence of metastatic disease was always based upon data obtained at post-mortem or biopsy except in the case of 10 patients with radiological signs of skeletal deposits. The liver, lungs and brain were examined in all patients submitted to post-mortem ; the spine was usually examined as well in these cases but other parts of the skeleton were rarely inspected unless the presence of bone metastases had been suspected during life. Metastases in other sites were not taken into account in the present study.

*Histological examination.*—After the selection of cases comprising this series had been completed, the sections of all the primary neoplasms were submitted to further histological examination. This work was undertaken by an independent observer who was unaware of the E.S.R. or the results of other data already considered. Each section was examined with reference to the following characteristics : (a) the degree of malignancy of the tumour ; (b) The degree of accumulation of lymphocytes in and around the neoplasm ; (c) the degree of fibrosis (i.e. stromal reaction) within and around the neoplasm ; (d) The amount of necrosis evident in the growth.

Although the degree of differentiation (or lack of differentiation) of neoplastic tissue was accepted as the main criterion of malignancy, other features such as the variation in the size and shape of the cells as well as the number of mitotic figures were also taken into account. Based on these criteria, the sections were divided into three grades. The presence of pleomorphism in some tumours, especially those arising in the bronchi, was responsible in a small proportion of cases for difficulty in allotting sections to an individual category. In this respect, Grade II was composed of a heterogeneous group of neoplasms which showed degrees of malignancy intermediate between the well differentiated tumours (Grade I) and the undifferentiated growths (Grade III). For this reason, statistical analysis designed to determine the association between the E.S.R. and the degree of malignancy was confined to the data obtained from the patients with neoplasms in which the degree of differentiation was sharply defined (i.e. Grades I and III).

The classification of the other histological characteristics was based upon the extent to which they were evident in the sections ; i.e. (1) no evidence of specific histological characteristic in section ; (2) minimal evidence, etc. ; (3) definite evidence etc. ; (4) very conspicuous evidence etc. Examination of the sections allotted to the second category was often inconclusive ; accordingly, these preparations, together with certain others derived from small biopsy specimens (examination of which was also unsatisfactory) were not taken into further account. For the purpose of statistical analysis, the sections allotted to the third and fourth categories were considered together ; this combination enabled the sections to be classified into two groups according to whether the specific histological characteristic was absent (first category) or present (third and fourth categories).

### RESULTS

TABLE II.—*Distribution of E.S.R. in Patients with Malignant Disease*

| Total number of patients | Number of patients in each category E.S.R. mm. in one hour | | | | |
|---|---|---|---|---|---|
| | 1–10 | 11–20 | 21–40 | 41–80 | >80 |
| 300 | 75 (25%) | 69 (23%) | 60 (20%) | 77 (26%) | 19 (6%) |

It is evident that one quarter of the total series of patients with malignant disease had a normal E.S.R., while in a further 23 per cent of the group there was only slight elevation of the E.S.R. These results may be contrasted with the high values observed in the majority of patients with multiple myelomatosis (Table III).

TABLE III.—*Distribution of E.S.R. in Patients with Multiple Myelomatosis*

| Total number of patients | Number of patients in each category E.S.R. mm. in one hour | | | | |
|---|---|---|---|---|---|
| | 1–10 | 11–20 | 21–40 | 41–80 | >80 |
| 25 | 1 | 1 | 4 | 8 | 11 |

It is apparent from Table IV that neoplasms originating in different sites of the body were associated with wide variations in the pattern of behaviour of the E.S.R. For example, the maintenance of normal values in over one third of the group of cases with gastric carcinoma was in marked contrast with the pronounced elevation of the E.S.R. seen in the majority of patients with carcinoma of the bronchus.

TABLE IV.—*E.S.R. and Site of Primary Neoplasm*

| Site of primary neoplasm | Total number of cases | Number of cases in each category E.S.R. mm. in one hour | | | | |
|---|---|---|---|---|---|---|
| | | 1–10 | 11–20 | 21–40 | 41–80 | >80 |
| Bronchus | 101 | 18 | 21 | 16 | 39 | 7 |
| Stomach | 51 | 18 | 15 | 11 | 7 | 0 |
| Colon | 27 | 3 | 7 | 12 | 4 | 1 |
| Pancreas | 15 | 5 | 5 | 3 | 2 | 0 |
| Breast without skeletal metastases | 14 | 13 | 0 | 1 | 0 | 0 |
| Breast with skeletal metastases | 14 | 2 | 1 | 2 | 6 | 3 |
| Brain | 13 | 7 | 4 | 2 | 0 | 0 |
| Uterus | 10 | 2 | 1 | 1 | 5 | 1 |
| Oesophagus | 8 | 1 | 4 | 1 | 2 | 0 |
| Ovary | 6 | 2 | 0 | 1 | 3 | 0 |
| Bone | 6 | 0 | 2 | 2 | 1 | 1 |
| Miscellaneous | 35 | 4 | 9 | 8 | 8 | 6 |
| Total | 300 | 75 (25%) | 69 (23%) | 60 (20%) | 77 (26%) | 19 (6%) |

Table V lists the cases of malignant disease in which the E.S.R. was greater than 100 mm. in one hour. It is evident that the development of extreme elevation of the E.S.R. was usually associated either with secondary infection or, more especially, with skeletal metastases.

TABLE V.—*Patients with an E.S.R.* > 100 *mm. in one hour*

| Site of primary neoplasm | Other features | E.S.R. (mm. in one hour) |
|---|---|---|
| Prostate | Skeletal metastases | 170 |
| Breast | Skeletal metastases | 135 |
| Breast | Skeletal metastases | 133 |
| Bladder | Cystitis and recto-vesical fistula | 130 |
| Bronchus | Infected atelectasis of right lower lobe of lung | 122 |
| Kidney (Grawitz tumour) | — | 120 |
| Bronchus | Septic bronchiectasis and skeletal metastases | 119 |
| Breast | Skeletal metastases | 118 |
| Uterus (cervix) | Skeletal metastases | 118 |
| Nasal sinus | Gross infection of adjacent bone | 113 |
| Colon | Pericolic abscess | 107 |
| Bronchus | Skeletal metastasis | 105 |
| Ewing's tumour of bone | — | 104 |

TABLE VI.—*E.S.R. and Fever*

| Fever | Total number of cases | Mean E.S.R. mm. in one hour | Standard error |
|---|---|---|---|
| Absent | 202 | 21·4 | 2·04 |
| Present | 98 | 35·3 | 3·78 |

The presence of a significant difference between the mean values shown in Table VI ($t = 3{\cdot}232 : P = <0{\cdot}01$) indicates that elevation of the E.S.R. was associated with the presence of fever.

TABLE VII.—*E.S.R. and Leucocytosis*

| Leucocytosis | Total number of cases | Mean E.S.R. mm. in one hour | Standard error |
|---|---|---|---|
| Absent . . | 207 . | 22·1 . | 2·09 |
| Present . . | 93 . | 32·8 . | 3·66 |

It may also be accepted that acceleration of the E.S.R. was associated with the presence of leucocytosis in that there was a significant difference between the mean values shown in Table VII ($t = 2{\cdot}541 : P = <0{\cdot}02$).

On the other hand, it is evident that the E.S.R. was not associated with the presence of anaemia as the respective mean values of the 176 patients with this complication (29·4 : S.E. = 1·89) and the 124 patients without anaemia (24·2 : S.E. = 2·54) did not differ significantly ($t = 1{\cdot}640 : P = >0{\cdot}05$).

Similarly, no correlation could be demonstrated between the E.S.R. and the development of loss of weight in as much as the respective mean values of the 185 patients with weight loss (28·5 : S.E. = 1·92) and the 115 patients without loss of weight (23·8 : S.E. = 2·68) were not significantly different ($t = 1{\cdot}424 : P = >0{\cdot}05$).

TABLE VIII—*E.S.R. and Secondary Infection*

(Patients with carcinoma of the bronchus)

| Secondary infection | Total number of cases | Mean E.S.R. mm. in one hour | Standard error |
|---|---|---|---|
| Absent . . | 50 | 22·3 . | 3·43 |
| Present . . | 51 . | 46·7 . | 4·23 |

The demonstration of a significant difference between the mean values shown in Table VIII ($t = 4{\cdot}477 : P = < 0{\cdot}001$) may be taken to indicate that elevation of the E.S.R. was associated with secondary infection in the group of patients with carcinoma of the bronchus.

TABLE IX.—*E.S.R. of Patients with Systemic Metastases*

| Site of metastases | Total number* of cases | Number of cases in each E.S.R. category E.S.R. mm. in one hour | | | | |
|---|---|---|---|---|---|---|
| | | 1–10 | 11–20 | 21–40 | 41–80 | >80 |
| Liver . . . | 58 . | 12 | 13 | 13 | 13 | 7 |
| Skeleton . . | 38 . | 5 | 5 | 6 | 14 | 8 |
| Lungs . . . | 19 . | 3 | 3 | 6 | 5 | 2 |
| Brain . . . | 16 . | 2 | 3 | 5 | 4 | 2 |

* The figures for the incidence of metastatic disease shown in this table are based upon certain criteria described under Methods.

Table IX indicates that the development of systemic metastases was more often than not associated with acceleration of the E.S.R. ; nevertheless, normal values were recorded in an appreciable proportion of cases, especially those with hepatic deposits. In a number of subjects, elevated rates could have been due either to the metastasis or to the primary growth particularly when the latter was associated with secondary infection (or to a combination of these factors). Furthermore, some patients developed metastases in more than one organ of the body. Accordingly, statistical analysis designed to assess the effect of metastases on the E.S.R. was confined to two selected groups of cases with secondary growths in the liver and skeleton respectively in which factors such as secondary infection and metastases in multiple sites could be excluded with reasonable certainty.

TABLE X.—*Mean E.S.R. of Selected Patients with and without Hepatic Metastases*

| Hepatic metastases | Total number of cases | Primary site of neoplasm | Mean E.S.R. mm. in one hour | Standard error |
|---|---|---|---|---|
| Absent | 13 | Stomach — 7 cases<br>Bronchus— 6 cases | 26·4 | 3·44 |
| Present | 11 | Stomach — 6 cases<br>Bronchus— 5 cases | 23·6 | 3·33 |

Since the mean E.S.R. of the selected group of cases with hepatic metastases was not significantly different from the mean value of the patients devoid of secondary deposits in the liver ($t = 0{\cdot}584 : P = >0{\cdot}05$), it would seem that the development of hepatic metastases did not contribute to the elevation of the E.S.R. in this group.

TABLE XI.—*Mean E.S.R. of Patients with and without Skeletal Metastases*

| Skeletal metastases | Total number of cases | Primary site of neoplasm | Mean E.S.R. mm. in one hour | Standard error |
|---|---|---|---|---|
| Absent | 13 | Breast | 6·7 | 0·80 |
| Present | 11 | ,, | 59·9 | 14·84 |

Table XI shows that the presence of skeletal metastases in a selected group of patients with carcinoma of the breast was associated with a very high mean E.S.R. ; this value differed significantly from the mean E.S.R. of a selected group of patients with mammary carcinoma in whom there was no evidence of osseous deposits ($t = 3{\cdot}580 : P = <0{\cdot}01$). It may be accepted, therefore, that the development of skeletal metastases was responsible for elevation of the E.S.R. in the first group of patients with carcinoma of the breast.

TABLE XII.—*Mean E.S.R. of Patients with and without Leuco-erythroblastic Anaemia*

| Leuco-erythroblastic anaemia* | Total number of cases | Mean E.S.R. mm. in one hour | Standard error |
|---|---|---|---|
| Absent | 21 | 56·8 | 7·29 |
| Present | 17 | 63·2 | 10·80 |

* All patients had evidence of skeletal metastases.

The absence of a significant difference between the respective mean values shown in Table XII ($t = 0{\cdot}491 : P = >0{\cdot}05$) shows that elevation of the E.S.R

in the patients with skeletal metastases (irrespective of the site of the primary neoplasm) was not due to the development of leuco-erythroblastic anaemia.

TABLE XIII.—*E.S.R. of Patients with Primary and Secondary Cerebral Neoplasms*

| Cerebral neoplasms | Total number of cases | Number of cases in each E.S.R. category (mm. in one hour) 1–10 | 11–20 | 21–40 | 41–80 | >80 |
|---|---|---|---|---|---|---|
| Primary | 13 | 7 | 4 | 2 | 0 | 0 |
| Secondary | 16 | 2 | 3 | 5 | 4 | 2 |

Table XIII shows that the E.S.R. was normal or only slightly elevated in the majority of patients with primary neoplasms of the brain in contrast to those with metastases in this site most of whom developed high values.

Data obtained from patients with bronchial carcinoma and other neoplasms in whom there was unequivocal evidence of secondary infection were not included in the subsequent statistical analyses of the histological data.

TABLE XIV.—*E.S.R. and Tumour Necrosis*

| Necrosis | Total number of cases | Mean E.S.R. mm. in one hour | Standard error |
|---|---|---|---|
| Absent | 112 | 22·3 | 3·55 |
| Present | 91 | 37·6 | 4·12 |

The presence of a significant difference between the mean values shown in Table XIV ($t = 2{\cdot}812 : P = <0{\cdot}01$) may be accepted as indicating that elevation of the E.S.R. was associated with the presence of tumour necrosis.

On the other hand, it may be concluded that no correlation existed between the E.S.R. and the degree of malignancy of neoplastic tissue for the respective mean values of the 69 patients with poorly differentiated growths, i.e. Grade III neoplasms (25·7 : S.E. = 3·40) and the 85 patients with well differentiated growths, i.e. Grade I neoplasms (20·8 : S.E. = 3·57) were not significantly different ($t = 0{\cdot}993 : P = >0{\cdot}05$).

There was also no association between the E.S.R. and the degree of lymphocytic accumulation in and around the neoplastic tissue in as much as the respective mean values of the 85 patients with growths showing this characteristic (22·7 : S.E. = 3·28) and the 60 patients with growths devoid of this feature (24·2 : S.E. = 2·98) did not differ to a significant extent ($t = 0{\cdot}338 : P = >0{\cdot}05$).

Similarly, it may be accepted that no correlation existed between the E.S.R. and the degree of fibrosis in and around the neoplastic tissue for the respective mean values of the 121 patients with growths displaying this feature (21·3 : S.E. = 1·99) and the 62 subjects with growths devoid of fibrosis (18·5 : S.E. =2·74) were not significantly different ($t = 0{\cdot}825 : P = >0{\cdot}05$).

It is evident from Table XV that the E.S.R. varied widely in the patients with jaundice.

### DISCUSSION

The results obtained in this study confirm the view that malignant disease is not always associated with rapid erythrocyte sedimentation. Nevertheless, the risk of overlooking malignancy, especially during its early stages, appears to be

TABLE XV.—*Distribution of E.S.R. in Patients with Jaundice*

| Site of neoplasm | Total number of patients | Number of patients in each category E.S.R. mm. in one hour: 1–10 | 11–20 | 21–40 | 41–80 | >80 |
|---|---|---|---|---|---|---|
| Hepatic metastases | 13 | 4 | 3 | 3 | 2 | 1 |
| Pancreas | 8 | 3 | 2 | 2 | 1 | 0 |
| Ampulla of Vater | 5 | 2 | 0 | 0 | 2 | 1 |
| Gall bladder | 1 | 0 | 0 | 1 | 0 | 0 |
| Total | 27 | 9 | 5 | 6 | 5 | 2 |

decidedly greater in patients in whom the E.S.R. is normal (Liljestrand and Olhagen, 1955). Sometimes, indeed, the symptoms of these patients may be regarded as psychogenic, especially if, in addition to a low E.S.R., physical examination and special investigations fail to reveal an organic lesion.

*Case* 1.—A man, aged 22 years, had complained for 2 years of flatulent dyspepsia associated with intermittent aching pain under the right costal margin; recently he had also vomited after meals. Physical examination was negative. A barium meal showed no evidence of an organic lesion, the haemoglobin value was 14·7 g. per cent and the E.S.R. was 4 mm. in one hour. His symptoms were considered to be of psychological origin especially as he had an aggressive manner. Nevertheless, his symptoms persisted and three months later he was seen again in the out-patient department. Physical examination was still negative and the E.S.R. was 7 mm. in one hour. However, a further barium meal showed evidence of an ulcerating neoplasm in the posterior wall of the body of the stomach. Subsequent laparotomy revealed that all the stomach, particularly the posterior aspect, was involved by a nodular infiltrating carcinoma which on section was shown to be poorly differentiated.

*Case* 2.—A woman, aged 39 years, had complained for 4 months of intermittent aching pain which was situated in the upper abdomen and radiated through to the back. Physical examination was negative. A barium meal and a chest X-ray were normal. The E.S.R. was 7 mm. in one hour. As she appeared to be hysterical and had the charge of an illegitimate child, she was referred to the psychiatric department. Five months later she still complained of abdominal pain although physical examination was negative, a further barium meal was normal and the E.S.R. was 6 mm. in one hour. Exploratory laparotomy revealed a mass in the head of the pancreas which on section was shown to be a well differentiated columnar cell adenocarcinoma of scirrhous type.

Diagnostic errors of this type are probably more likely to occur in connection with malignant tumours of the abdominal organs; in these cases, the symptoms are sometimes vague and ill defined and, as Case 1 illustrates, anaemia and radiological changes are not invariably present even if the alimentary tract is involved. However, the discovery of a normal E.S.R. may also be misleading in the event of malignancy developing elsewhere in the body.

*Case* 3.—A man, aged 53 years, had for 2 months noticed "cold sensations in the stomach" especially after drinking beer. He was otherwise symptom free. Physical examination was negative. A chest X-ray and barium meal were normal and the E.S.R. was 4 mm. in one hour. However, a further chest X-ray 10 weeks later showed an opacity in the left upper lobe; the E.S.R. was still normal at 6 mm. in one hour. During the next month his condition rapidly deteriorated and he died after developing bronchopneumonia. Post-mortem revealed a left upper lobe bronchial carcinoma which on section was shown to be highly anaplastic.

It is apparent, therefore, that only too often the E.S.R. is unaffected in the very case of malignant disease in which an elevated value would be of considerable diagnostic significance. Moreover, even when abnormal values were recorded in the present series, the degree of elevation was sometimes comparatively slight. The behaviour of the E.S.R. in these cases was certainly very different from that observed in the series of patients with multiple myelomatosis, most of whom developed extremely high sedimentation rates. It is of interest that in this disorder, in contrast to malignant disease, there is often a lengthy preclinical phase characterised by gross changes in the serum globulin fractions together with a very rapid E.S.R. (Liljestrand and Olhagen, 1955).

It is evident, however, that the limitations associated with measurement of the E.S.R. in malignant disease are not confined to its use as a screening test; clearly, it is also unreliable in providing an index of the clinical condition of these patients as well as in denoting the degree and extent of the neoplastic process. For example, no relationship could be demonstrated between the E.S.R. and the presence of either anaemia of loss of weight. In this connection, Terry (1950) and Westergren (1957) have suggested that the development of hypochromic anaemia, such as is commonly found in malignant disease of the gastro-intestinal tract, may actually retard the E.S.R., and Li (1943) has observed that the E.S.R. sometimes falls in malignancy as a result of extreme cachexia. Certainly, the contention of Rubin (1927) that the E.S.R. closely conforms with the clinical appearance of the patient in malignant disease could not be substantiated in the majority of those in the present series even though there were special groups of cases, notably those with secondary infection or skeletal metastases, for which this thesis usually held good. It is apparent, too, that there was no evidence of any correlation between the E.S.R. and the degree of malignancy of neoplastic tissue, as determined by histological examination; furthermore, the E.S.R. appeared to be unrelated to the natural history of malignant disease (although statistical data were not obtained in regard to this aspect of the study, information as to duration of illness and length of survival, etc., was available in respect of almost all cases). Even the presence of systemic metastases, with the exception of those which developed in the skeletal system, often failed to affect the sedimentation rate. It appears, therefore, that measurement of the E.S.R. is by no means a satisfactory method of appraising the prognosis of patients with malignant disease; furthermore, it is not often likely to be of value in deciding whether a surgical operation is feasible.

Although the E.S.R. remained within normal limits in an appreciable proportion of patients in the present series, more often than not there was some elevation. In the majority of these cases, such knowledge was of little more than academic interest in as much as the presence of malignant disease was readily confirmed by other means. In a number of patients, however, in whom this diagnosis was less easily achieved, the discovery of an increased E.S.R. proved to be of considerable importance.

*Case* 4.—A man, aged 61 years, presented with a history of haemoptyses for the previous 2 weeks. Physical and radiological examination of the chest as well as bronchoscopy revealed no evidence of disease. The E.S.R. was 22 mm. in one hour. The patient was kept under observation; during the next 10 weeks the

E.S.R. varied between 19 and 27 mm. in one hour. At the end of this period, a further chest X-ray was still normal but a second bronchoscopy revealed a carcinoma of the right main bronchus (biopsy showed an oat cell neoplasm).

Thus, as Strasser and Weiser (1954) have observed, the presence of an elevated E.S.R. in a patient with focal symptoms frequently provides a clear warning that malignant disease has developed; even if subsequent investigations prove negative, the maintenance of a rapid rate may indicate that this is in fact the correct diagnosis. It is important to recognise, too, that it is often the persistence of an elevated value in these cases rather than the degree of elevation which should be regarded with suspicion.

The present results suggest that determination of the E.S.R. is likely to find special application in the diagnosis of patients with skeletal metastases most of whom showed high values. For example, it is probably wise to measure the E.S.R. periodically in patients who have received treatment for neoplasms which have a special tendency to metastasise within the skeleton.

*Case* 5.—A woman, aged 33 years, underwent a radical mastectomy for a mammary carcinoma which was shown to be highly undifferentiated on histological examination. The E.S.R. was 4 mm. in one hour. Eighteen months later she re-attended hospital with a history of constant pain in the lower part of the back for the previous 6 weeks. Physical examination was negative and a series of X-rays of the lumbo-sacral spine and pelvis was normal. A blood count revealed a haemoglobin value of 13·2 g. per cent and a white cell count of 6,800 per c.mm. (the blood film was normal). However, the E.S.R. stood at 78 mm. in one hour. She was treated symptomatically with analgesics. Seven weeks later a further series of X-rays showed the presence of several osteolytic secondary deposits in the pubic rami and sacrum. The E.S.R. was now 90 mm. in one hour. A course of deep X-ray therapy to the pelvis was given with much relief of pain.

Furthermore, discovery of a raised E.S.R. in a middle-aged or elderly patient with skeletal pain should prompt a thorough search for a primary malignant tumour even in the absence of anaemia or radiological changes.

*Case* 6.—A man, aged 60 years, presented with a history of dull pain in the lower part of the back for 2 months. He was otherwise symptom free. Physical examination was negative and a series of X-rays of the spine, pelvis, and chest was normal. The E.S.R. was 35 mm. in one hour. Two months later the patient reported that the pain in the back was worse although physical examination remained negative. The haemoglobin value was 14·2 g. per cent and the white cell count was 7,800 per c.mm. (the blood film was normal). As before, the spine, pelvis and chest showed no radiological abnormality. However, the E.S.R. was still raised at 40 mm. in one hour. At this stage, examination of a bronchoscopic biopsy of the right lower lobe bronchus revealed an oat cell carcinoma. At post-mortem 2 months later, numerous metastatic deposits were found in the lumbar spine and pelvis.

In this connection, Wolfson, Reznick and Gunther (1941) suggested that an early diagnosis of spinal metastases could usually be made, despite the lack of radiological signs, if radicular pain was accompanied by an elevated E.S.R. and an increase in the serum alkaline phosphatase level. Wolfson and his colleagues also emphasized the rapidity with which a high E.S.R. appears in patients with osseous metastases in contrast to the general blood picture which frequently remains unaltered until the later stages of the illness.

The present study also suggests that measurement of the E.S.R. is sometimes likely to be useful in differentiating between primary and secondary cerebral neoplasms for whereas the majority of patients with primary lesions of the brain maintained normal or only slightly elevated values, most of those with metastatic growths in this site were observed to have developed rapid sedimentation. Klingman, Laidlaw and Spotnitz (1940) and Elliott, Hughes and Turner (1952) likewise considered that acceleration of the E.S.R. usually betokened the metastatic origin of a cerebral neoplasm, while Gram (1929) had previously contended that the E.S.R. was not elevated in patients with primary tumours of the brain. Nevertheless, this contrast is not always evident ; thus not only may cerebral metastases be associated with a normal E.S.R., especially if they originate in neoplasms devoid of secondary infection (personal observation), but also, according to Klingman and his colleagues, rapid sedimentation occasionally develops in patients with primary growths of the glioblastoma multiforme type.

One of the fastest sedimentation rates in the present series was recorded in a patient from whom a hypernephroma was subsequently removed ; the other two patients with this type of renal neoplasm also showed elevated values (admittedly, though, one of the latter subjects had developed skeletal deposits as well). Although the E.S.R. is not always accelerated in these cases (Olovson, 1946), it appears that the Grawitz tumour is one of the few examples of malignant disease which may give rise to fever and rapid erythrocyte sedimentation in the absence of local symptoms and signs (Johnsson, 1954) ; accordingly, the discovery of a high E.S.R. of unknown origin may occasionally necessitate complete examination of the renal tract including careful radiological studies.

It is apparent, therefore, that determination of the E.S.R. sometimes proves to be of value in the diagnosis and appraisal of patients with malignant disease, despite its evident limitations in this respect. It has to be remembered, of course, that although the E.S.R. is frequently increased in cancer, other conditions which often simulate malignant disease may also be associated with elevated rates. These include systemic disorders such as tuberculosis and the lymphomas as well as, sometimes, local lesions as exemplified by simple gastric ulcers (Britton, 1936 ; personal observation), diverticulitis (Britton, 1936), cerebral abscesses and subdural haematomas (Whitby and Britton, 1957) and even, occasionally, uterine fibroids, cervical erosions and simple ovarian cysts (Walton, 1933 ; Li, 1943 ; personal observation).

Since it is generally accepted that inflammation, irrespective of aetiology, is one of the main pathological processes responsible for elevation of the E.S.R., it is not surprising that an association could be demonstrated between rapid sedimentation and the presence of fever and leucocytosis. Even so, of these three manifestations of disease, acceleration of the E.S.R. was the one most often evident in this study. Scott (1938), too, considered that the sedimentation rate covered a wider field than the temperature chart especially as he found it to be frequently elevated in infection in the absence of pyrexia, and Gregg and Allen (1939) concluded that measurement of the E.S.R. was more reliable than estimation of the white cell count in denoting the degree of tissue destruction. In the present series, secondary infection was clearly often responsible for the development of an inflammatory reaction, especially in the patients with bronchial carcinoma ; in this group, there was a close relationship between the E.S.R and pulmonary sepsis. Kourilsky, Decroix and Duwoos (1952) likewise showed that the highest sedimentation rates in lung cancer usually occur in patients with bronchial obstruction and pulmonary collapse. However, secondary infection may also have contributed to the development of elevated values in a number of other patients in the present series particularly those with neoplasms arising in sites such as the colon, uterus and bladder. Certainly the development of ulceration, with the exception of gastric growths, was almost always associated with some acceleration of the E.S.R. In this connection, Alvarez (1953) emphasised that the passage of bacteria through friable tumours was undoubtedly one of the most important factors responsible for rapid sedimentation in malignant disease. Nevertheless, it is remarkable that in the series under review the E.S.R. was often unaffected in patients with gastric neoplasms even though examination of these growths usually revealed an ulcerous or polypoid type of lesion. Possibly, however, the development of secondary infection in these growths is largely prevented by the bactericidal effect of gastric juice. On the other hand, according to Stemmler (1925) the maintenance of a normal E.S.R. in many patients with carcinoma of the stomach is primarily due to the absence of necrosis in most of these growths.

It is evident, however, that secondary infection is not the only factor responsible for elevation of the E.S.R. in malignant disease. The present study amply confirmed, in fact, the widely held impression as to the role of tumour necrosis in these cases. Furthermore, although the statistical evidence of a relationship between the E.S.R. and tumour necrosis was obtained in respect of a group of patients apparently devoid of sepsis, a similar effect may well have prevailed in some of those with secondary infection (e.g. the group with bronchial carcinoma); indeed, it is well recognised that sepsis and tissue destruction are sometimes closely related. In this connection, Gram (1929) maintained that the E.S.R. is often influenced in disease by several factors simultaneously.

The results of the present study are of interest in the light of the observations reported by Liljestrand and Olhagen (1955). They showed that although malignant disease is not usually associated with a specific blood protein pattern, increased levels of the fibrinogen and $alpha_2$ globulin fractions are sometimes to be found in the plasma of patients with high sedimentation values, particularly those with neoplasms in organs such as the lungs, pancreas, gonads and kidney; as these workers emphasized, similar alterations in the plasma proteins are seen in numerous other conditions characterized by infection and/or tissue destruction. Whether or not other pathological processes also influence the E.S.R. to any significant extent in malignant disease was not revealed by the present study. Certainly, however, the mechanism responsible for rapid sedimentation in some of the patients with skeletal metastases was by no means clearly established for although this form of malignancy was usually associated with a conspicuous inflammatory reaction, as judged by the degree of fever and leucocytosis, there were a number of cases in which neither secondary infection nor tumour necrosis appeared to be implicated. It is therefore of interest that according to Böttiger (1957) elevation of the E.S.R. sometimes develops in patients with hypernephromas devoid of both these features. Recently, Böttiger and Ivemark (1959) have claimed that a close connection exists between the cellular differentiation of such neoplasms and the development of fever and high sedimentation values in as much as these signs are usually associated with tumours composed mainly of clear cells rather than those of granular type; in a further report, Böttiger (1960) has suggested that the clear cells (the cytoplasm of which usually reacts to the periodic acid-Schiff stain) may produce a factor which is capable of inducing glycoprotein synthesis.

Evidence derived from this study suggests that the E.S.R. is not influenced by the presence of metastases in the liver ; more probably it is affected in these cases by other factors such as the characteristics of the primary growth (e.g. the presence of secondary infection) or the co-existence of metastases in the bones. It is pertinent to remember, in this connection, that hepato-cellular function is adequate in patients with carcinomatosis of the liver, and in the majority of them there is no gross alteration in the plasma protein levels (Sherlock, 1958). The results of the present investigation are in agreement with those of Walton (1933) who reported that the E.S.R. is often normal in these patients. On the other hand, Rosenthal and Blowstein (1929) and Sherlock (1958) concluded that metastases in this site do in fact usually give rise to rapid sedimentation ; indeed, the former workers felt that measurement of the E.S.R. would prove to be extremely useful in determining whether jaundice was due to malignant disease since in their series elevated values were almost always found in patients with neoplasms obstructing the outflow of bile in contrast to those with infective hepatitis in whom normal rates were maintained. (Sherlock has affirmed, however, that the E.S.R. is increased during the pre-icteric and convalescent phases of virus hepatitis). Lipp and Aaron (1942) likewise observed that obstructive jaundice of malignant origin (both hepatic and extra-hepatic) was invariably accompanied by high sedimentation rates. However, the results of the present study suggest that it is probably unwise to place too much reliance on the E.S.R. in these cases for normal values were recorded not only in some of the patients with hepatic metastases but also in a number of those with tumours originating in the pancreas and ampulla of Vater.

It is well recognised that patients with malignant neoplasms, especially those with hypernephromas, occasionally present with polycythaemia (Romagny, Viallier and Blanchard, 1941 ; Conley, Cowal and D'Antonio, 1957 ; Damon, Holub, Mellicow and Uson, 1958). Nevertheless, the presence of malignant disease is not always immediately suspected in these cases. According to Drivsholm (1960), however, an E.S.R. greater than 1 mm. in one hour (Westergren) in a patient with a high red cell count should direct attention to the possible existence of a hypernephroma in as much as primary polycythaemia is usually associated with a value of 1 mm. or less. In the present series, none of the patients with hypernephromas or other neoplasms developed polycythaemia. It is of interest, though, that the E.S.R. stood at 3 mm. in the patient discussed by Drivsholm. However, other reports (Videbaek, 1950 ; Forssell, 1958) indicate that the E.S.R. does not invariably exceed 1 mm. during the early stages of the hypernephroma-polycythaemia syndrome even though it may do so during the subsequent course of the disease.

### SUMMARY

The behaviour of the erythrocyte sedimentation rate (E.S.R.) was studied in a series of 300 patients with malignant disease.

Twenty-five per cent of the patients were found to have an E.S.R. within the normal range, i.e. 1–10 mm. in one hour (Westergren) ; a further 23 per cent developed an E.S.R. within the " slightly elevated " range, i.e. 11–20 mm. in one hour. Only 4 per cent of the cases had an E.S.R. greater than 100 mm. in one hour. These results were contrasted with those obtained in 25 patients with multiple myelomatosis, 23 of whom developed an E.S.R. more than 20 mm. in one hour.

The distribution of the E.S.R. varied widely according to the site of the primary neoplasm. For example, 65 per cent of the patients with gastric carcinoma were shown to have an E.S.R. within the 1–20 mm. range in contrast to only 39 per cent of those with carcinoma of the bronchus.

Elevation of the E.S.R. was closely associated with the presence of fever and leucocytosis. On the other hand, no correlation could be demonstrated between the E.S.R. and the presence of either loss of weight or anaemia.

Secondary infection and tumour necrosis, either separately or in combination, appeared to be the main pathological factors responsible for the development of rapid sedimentation in the present series. No relationship, however, could be shown to exist between the E.S.R. and the degree of malignancy of neoplastic tissue, as determined by histological examination ; furthermore, there was no evidence that the E.S.R. was influenced either by the amount of fibrosis within the tumour or by the degree of lymphocytic accumulation in and around the growth.

The development of skeletal metastases clearly accounted for elevation of the E.S.R. in a number of patients in this series ; secondary deposits in the bones were evident in no less than 7 of the 13 patients with an E.S.R. greater than 100 mm. in one hour. On the other hand, the E.S.R. was not apparently affected by the presence of hepatic metastases. Rapid sedimentation was observed in the majority of patients with cerebral metastases in contrast to those with primary neoplasms of the brain most of whom were found to have normal values.

The development of jaundice was associated with wide variations in the E.S.R.

The present study revealed that measurement of the E.S.R. is often unreliable in malignant disease not only as a screening test but also in providing an index of the severity and extent of the neoplastic process ; nevertheless, evidence is presented which suggests that this procedure should in fact sometimes prove to be of value in the diagnosis and appraisal of these patients.

I wish to thank the physicians of Charing Cross Hospital for allowing me to study their patients ; Professor W. St.C. Symmers for his encouragement ; Dr. J. H. Shore, who, as an independent observer, undertook the histological examination of the sections ; and Dr. G. W. Bisset and Mr. R. A. Husain for help with the statistical analysis.

### REFERENCES

Agnor, E. B.—(1940) *Ann. intern. med.*, **14**, 774.
Alston, J. M.—(1946) *Practitioner*, **157**, 23.
Alvarez, W. C.—(1953) Quoted by Lipschutz (1953).
Bannick, E. G., Gregg, R. O. and Guernsey, C. M.—(1937) *J. Amer. med. Ass.*, **109**, 1257.
Böttiger, L. E.—(1957) *Acta med. scand.*, **156**, 477.—(1960) *Ibid.*, **167**, 455.
*Idem* and Ivemark, B. I.—(1959) *J. Urol.*, **81**, 512.
Bouton, S. M.—(1938) *J. Lab. clin. Med.*, **23**, 519.
Britton, C. J. C.—(1936) *N.Z. med. J.*, **35**, 310.
Conley, C. L., Cowal, J. and D'Antonio, J.—(1957) *Johns Hopk. Hosp. Bull.*, **101**, 63.

CUTLER, J. W.—(1932) *Amer. J. med. Sci.*, **183**, 643.
*Idem*, PARK, F. R. AND HERR, B. S.—(1938) *Ibid.* **195**, 734.
DAMON, A., HOLUB, D. A., MELLICOW, M. M. AND USON, A. C.—(1958) *Amer. J. Med.*, **25**, 182.
DRIVSHOLM, A.—(1960) *Brit. med. J.*, ii, 1063.
ELLIOTT, F. A., HUGHES, E. AND TURNER, J. W. A.—(1952) ' Clinical Neurology '. London (Cassell and Co.).
FAHRAEUS, R.—(1921) *Acta med. scand.*, **55**, 1.
FEARNLEY, G. R.—(1957) *Practitioner*, **178**, 705.
FORSSELL, J.—(1958) *Acta med. scand.*, **161**, 169.
GRAM, H. C.—(1929) *Ibid.*, **70**, 242.
GREGG, R. O. AND ALLEN, E. G.—(1939) *N.Y. St. J. Med.*, **39**, 2192.
JOHNSON, A. S.—(1939) *New Engl. J. Med.*, **220**, 823.
JOHNSSON, S.—(1954) *Nord. med.*, **52**, 1434.
KLINGMAN, W. O., LAIDLAW, R. W. AND SPOTNITZ, H.—(1940) *N. Y. St. J. Med.*, **40**, 117.
KOURILSKY, R., DECROIX, G. AND DUWOOS, H.—(1952) *J. franç. Méd. Chir. thor.*, **6**, 263.
LI K. Y. Y.—(1943) *Amer. J. Obstet. Gynec.*, **46**, 381.
LILJESTRAND, A. AND OLHAGEN, B.—(1955) *Acta med. scand.*, **151**, 425.
LIPP, W. F. AND AARON, A. H.—(1942) *N.Y. St. J. Med.*, **42**, 1951.
LIPSCHUTZ, E. W.—(1953) *Rev. Gastroent.*, **20**, 725.
NICHOLS, D. R.—(1945) *Med. Clin. N. Amer.*, **29**, 936.
OLOVSON, T.—(1946) *Acta chir. scand.*, **93**, 503.
POOLE, J. C. F. AND SUMMERS, G. A. C.—(1952) *Brit. med. J.*, i, 353.
REICHEL, H.—(1936) *Med. Klinik*, **32**, 1769.
ROMAGNY, G., VIALLIER, J. AND BLANCHARD, H.—(1941) *Lyon méd.*, **166**, 785.
ROSENTHAL, N. AND BLOWSTEIN, M. I.—(1929) *J. Lab. clin. Med.*, **14**, 464.
RUBIN, E. H.—(1927) *Amer. J. med. Sci.*, **174**, 680.
SCOTT, E.—(1938) *Brit. med. J.*, i, 722.
SHERLOCK, S.—(1958) ' Diseases of the liver and biliary system '. Oxford (Blackwell).
STEMMLER, W.—(1925) *Arch. klin. Chir.*, **137**, 705.
STRASSER, A. AND WEISER, F.—(1954) *Klin. Med.*, **9**, 433.
TERRY, R.—(1950) *Brit. med. J.*, ii, 1296.
VICKERS, D. M. AND DURYEE, R.—(1932) *J. Lab. clin. Med.*, **18**, 260.
VIDEBAEK, A.—(1950) *Acta. med. scand.*, **138**, 239.
WALTON, A. C. R.—(1933) *Quart. J. Med.*, **2**, 79.
WESTERGREN, A.—(1957) *Triangle*, **3**, 20.
WHITBY, L. E. H. and BRITTON, C. J. C.—(1957) ' Disorders of the Blood '. London (Churchill).
WOLFSON, S. A., REZNICK, S. AND GUNTHER, L.—(1941) *J. Amer. med. Ass.*, **116**, 1044.


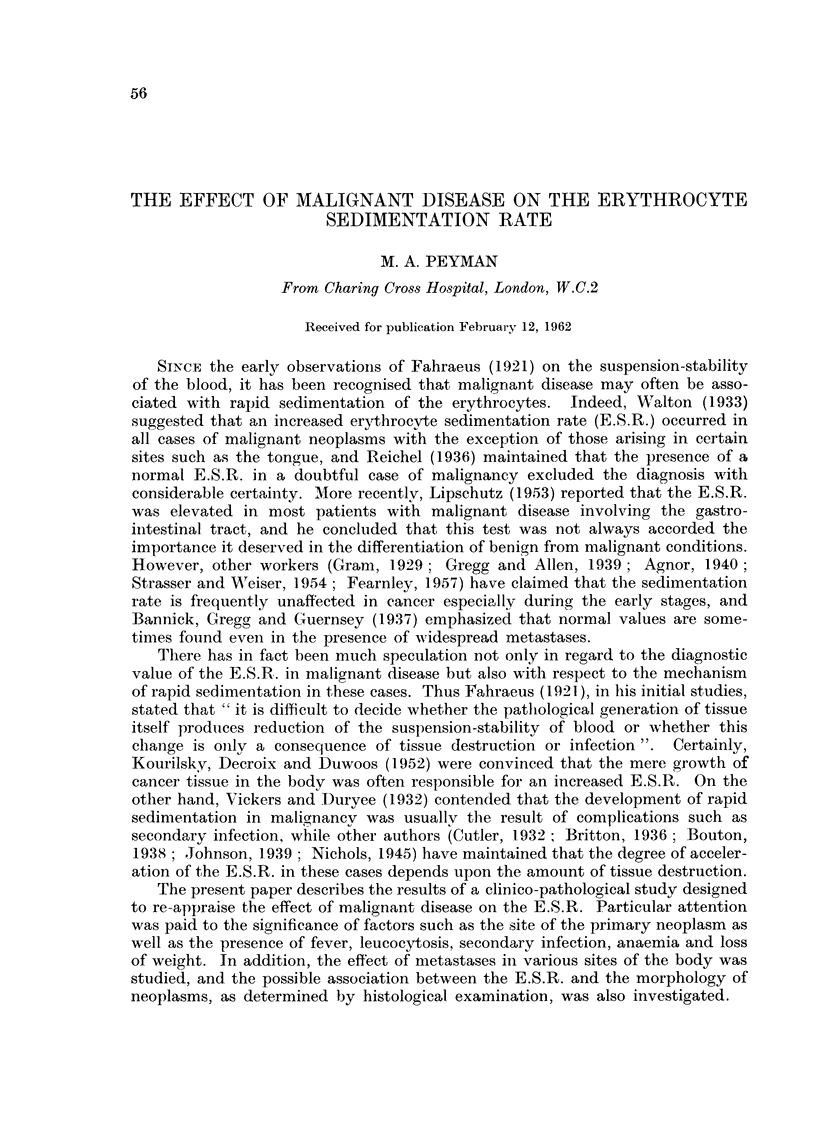

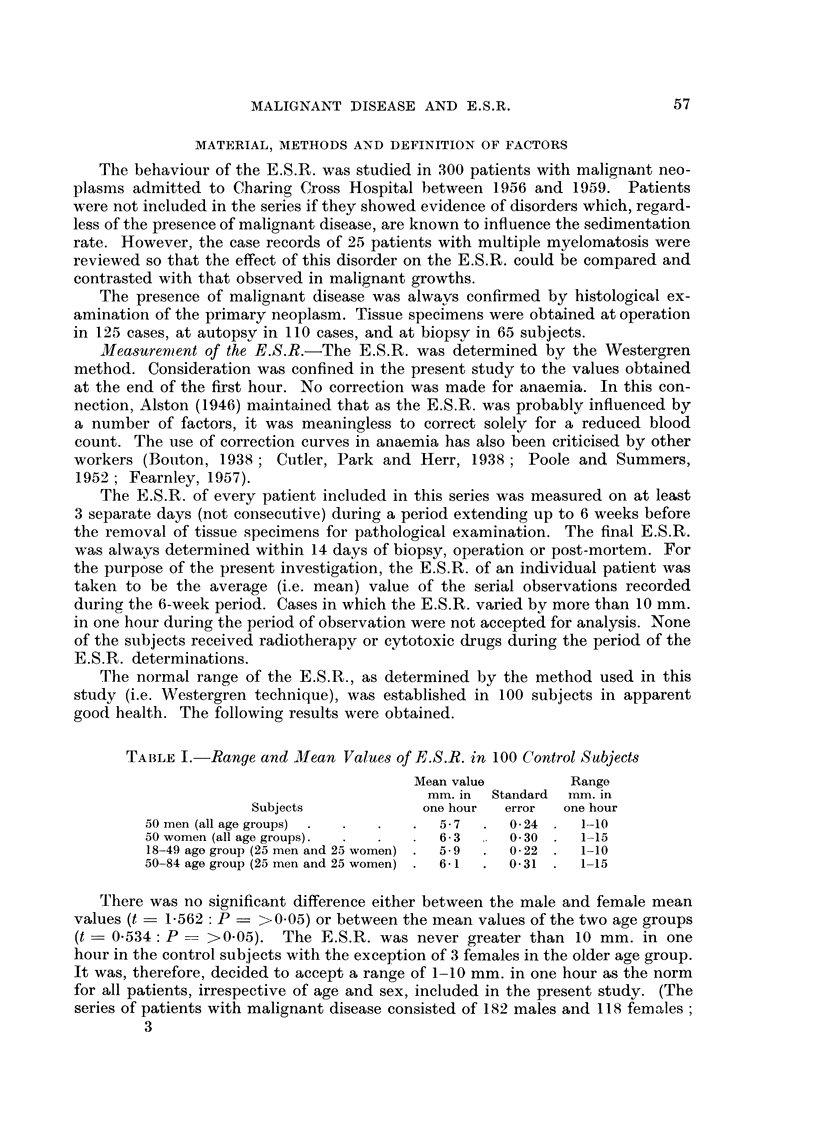

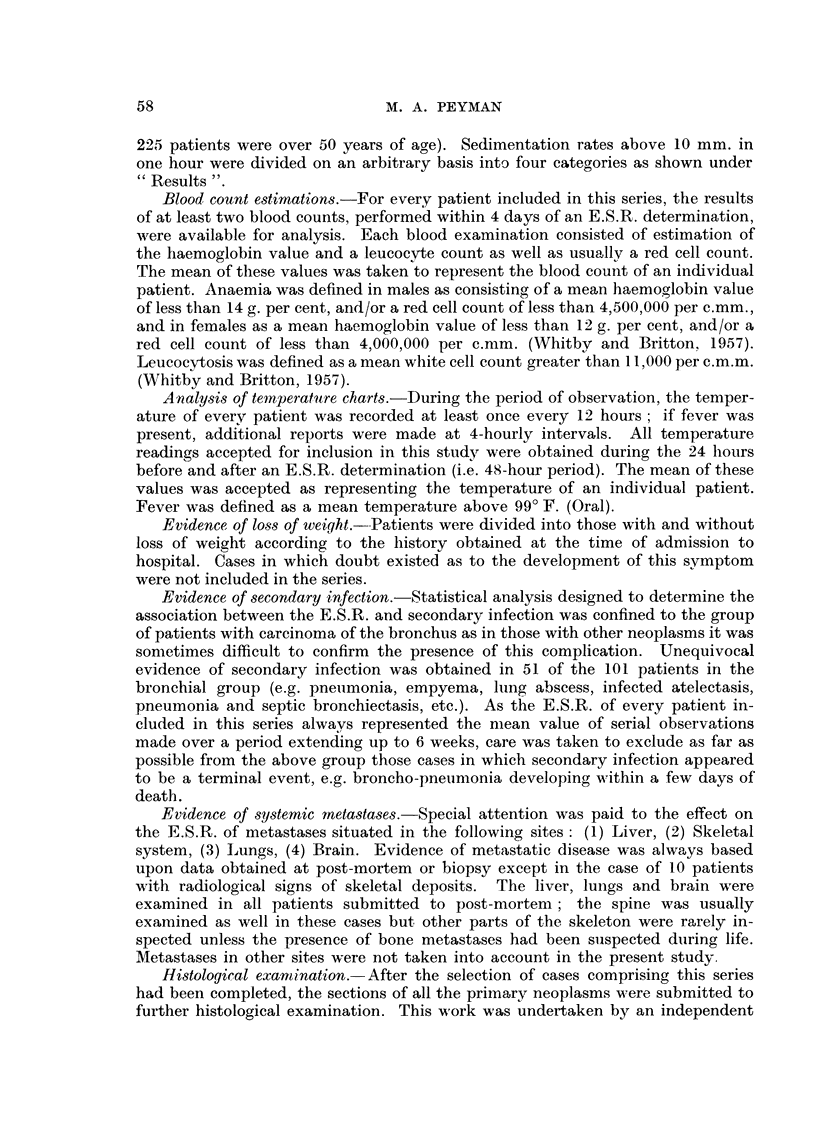

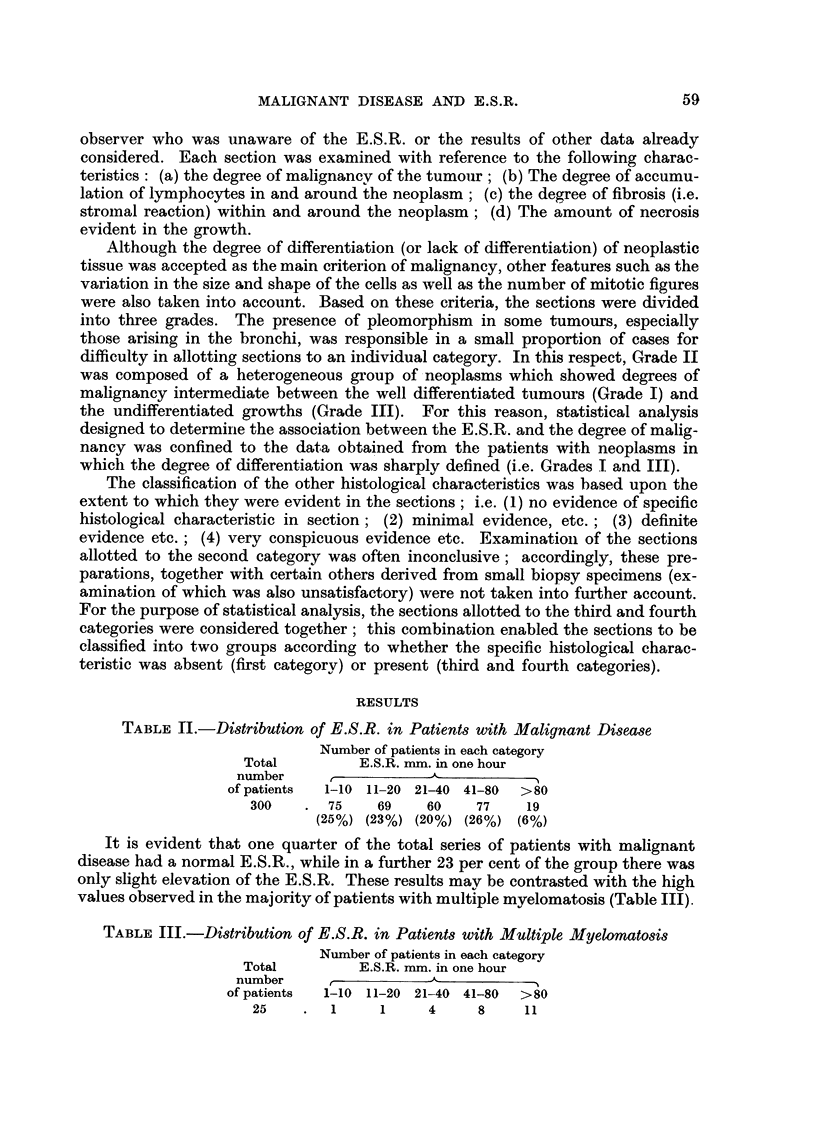

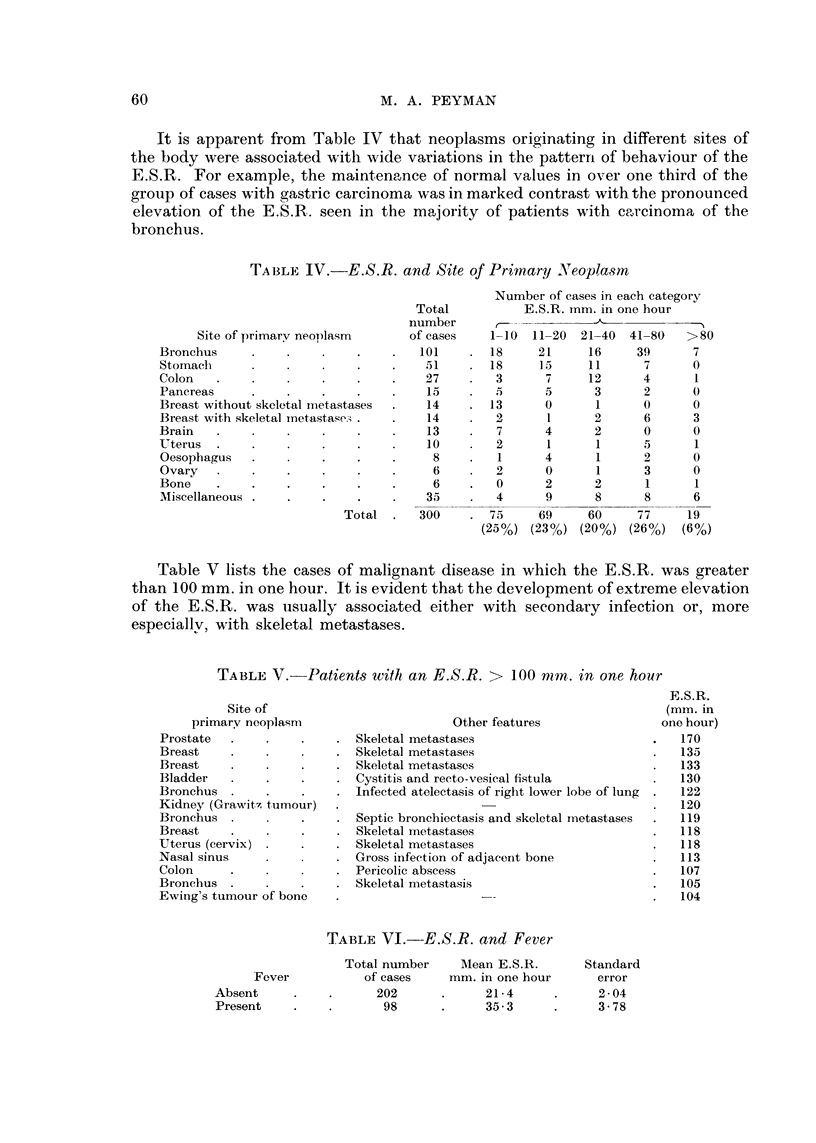

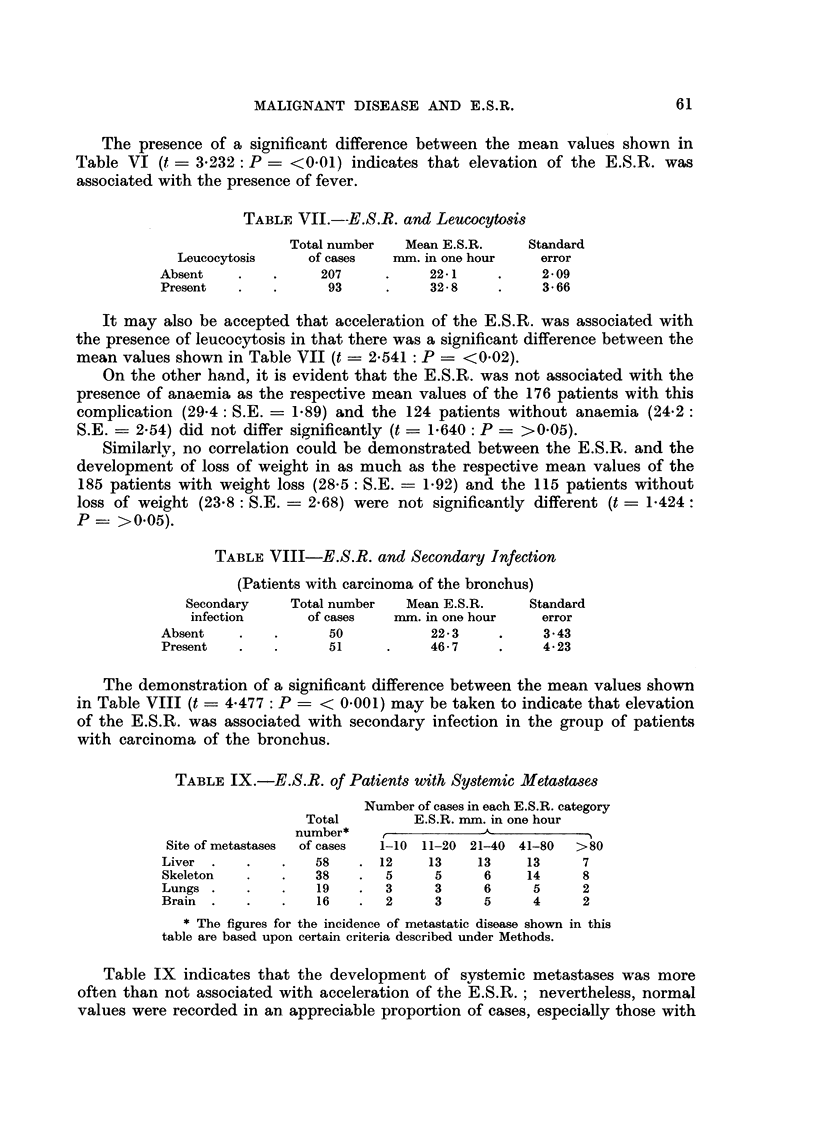

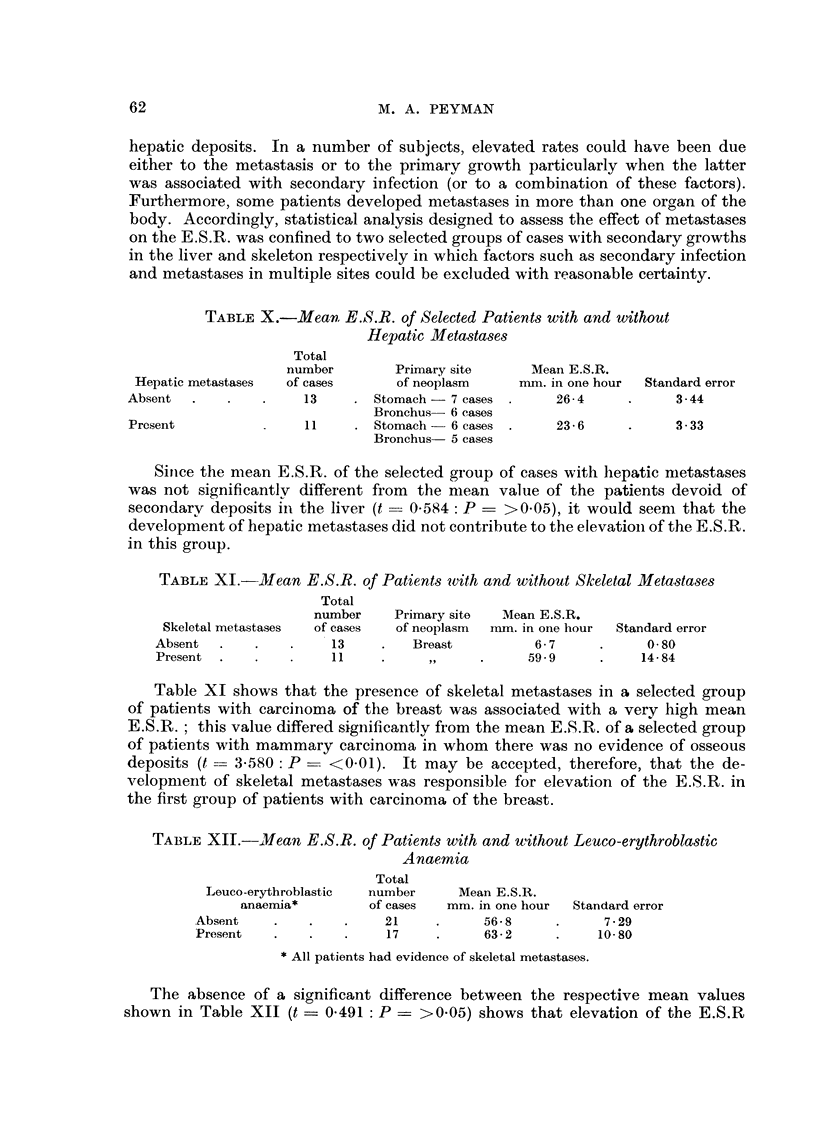

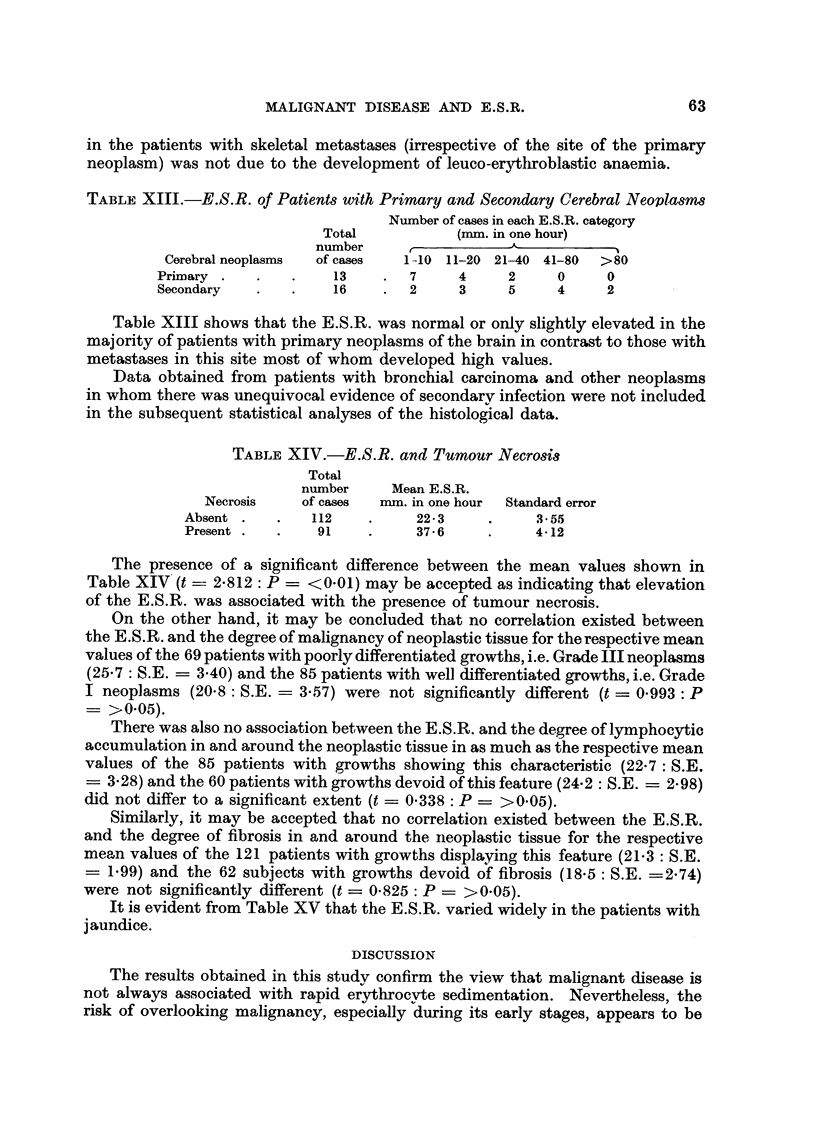

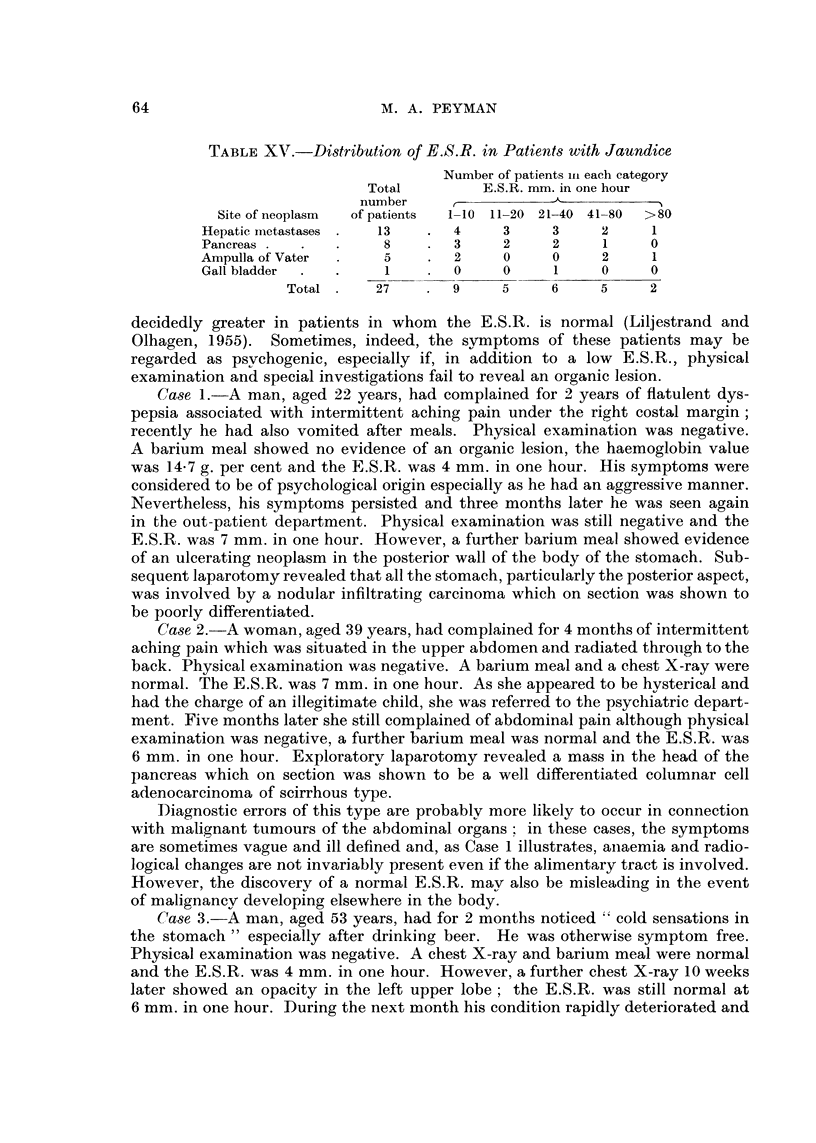

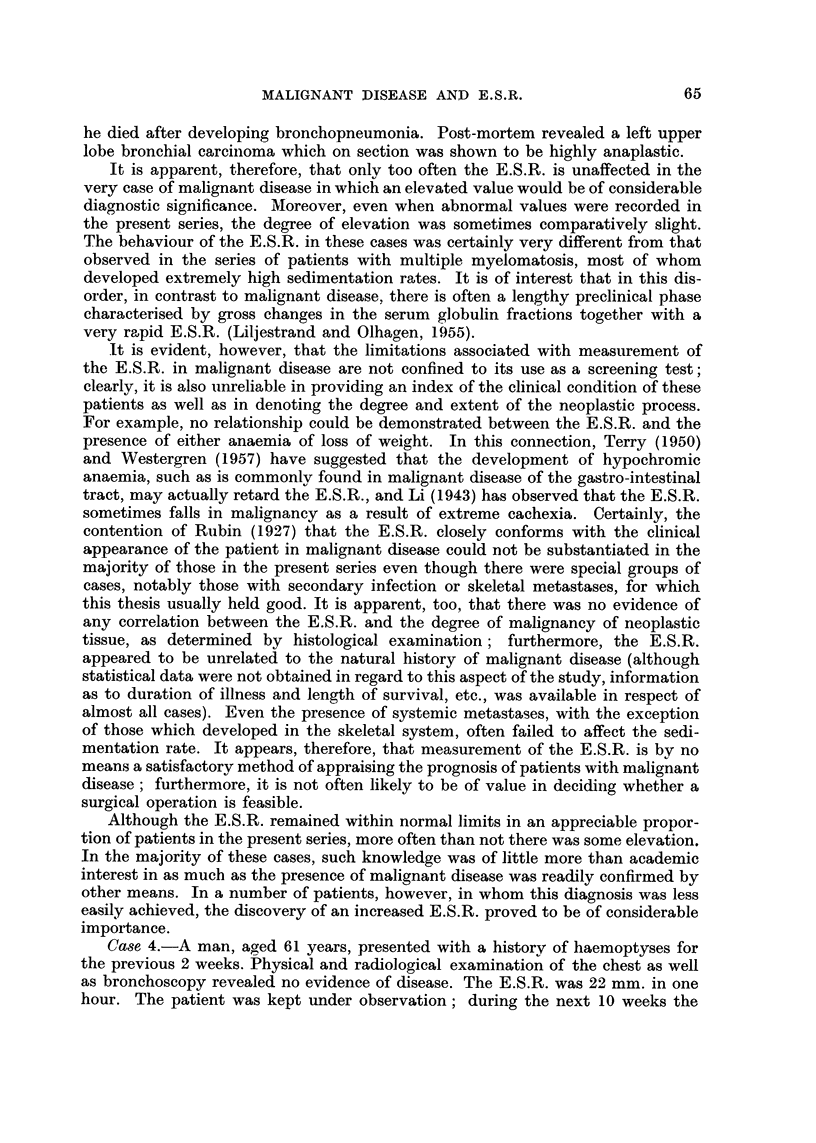

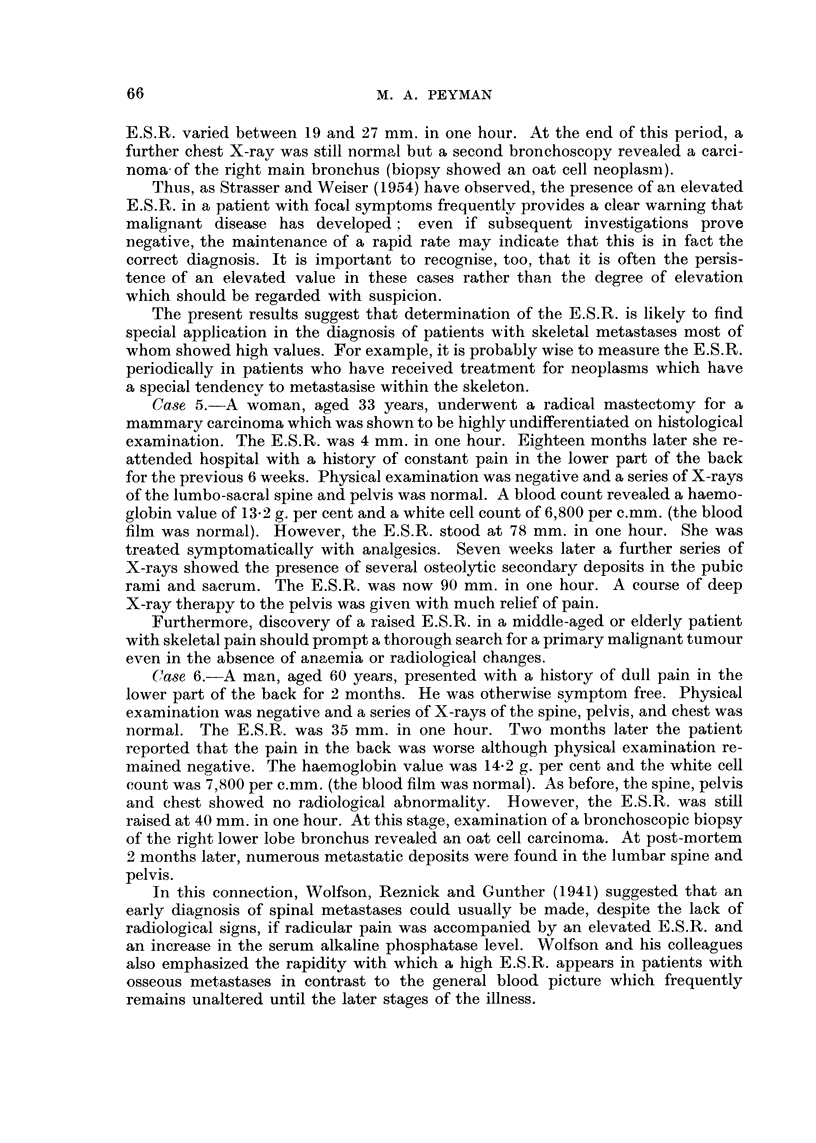

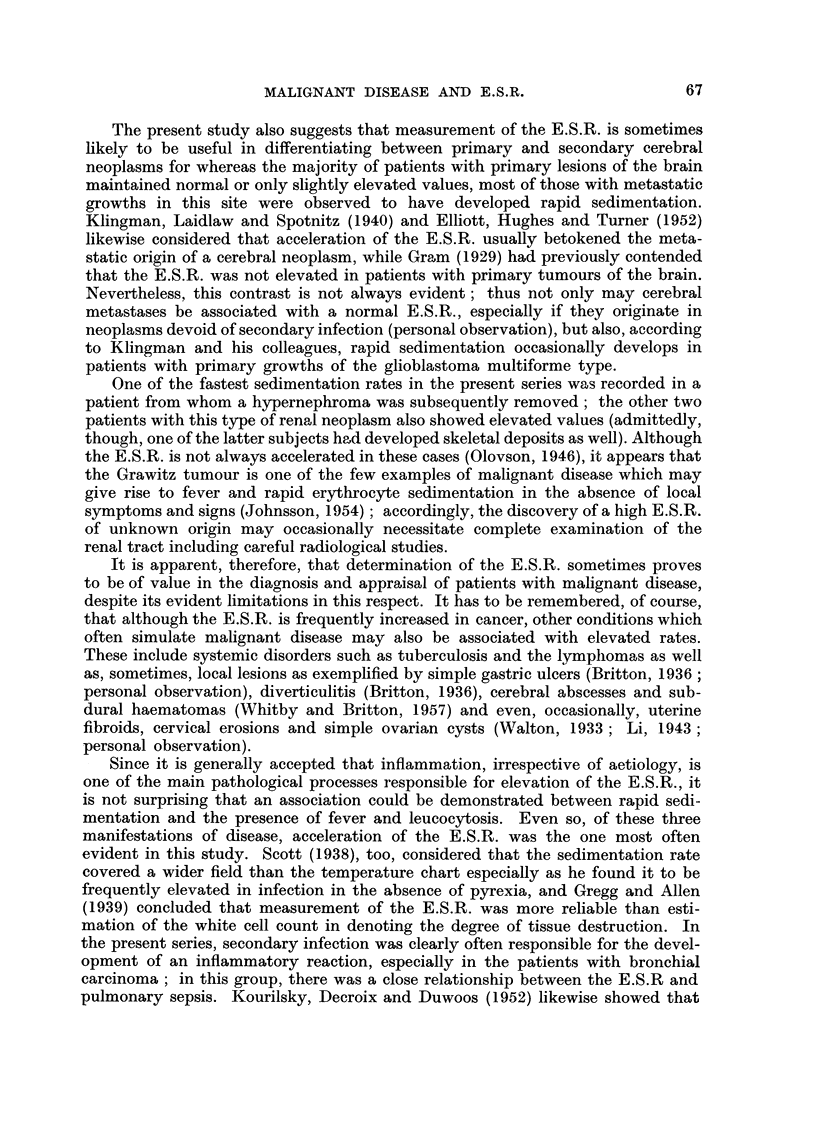

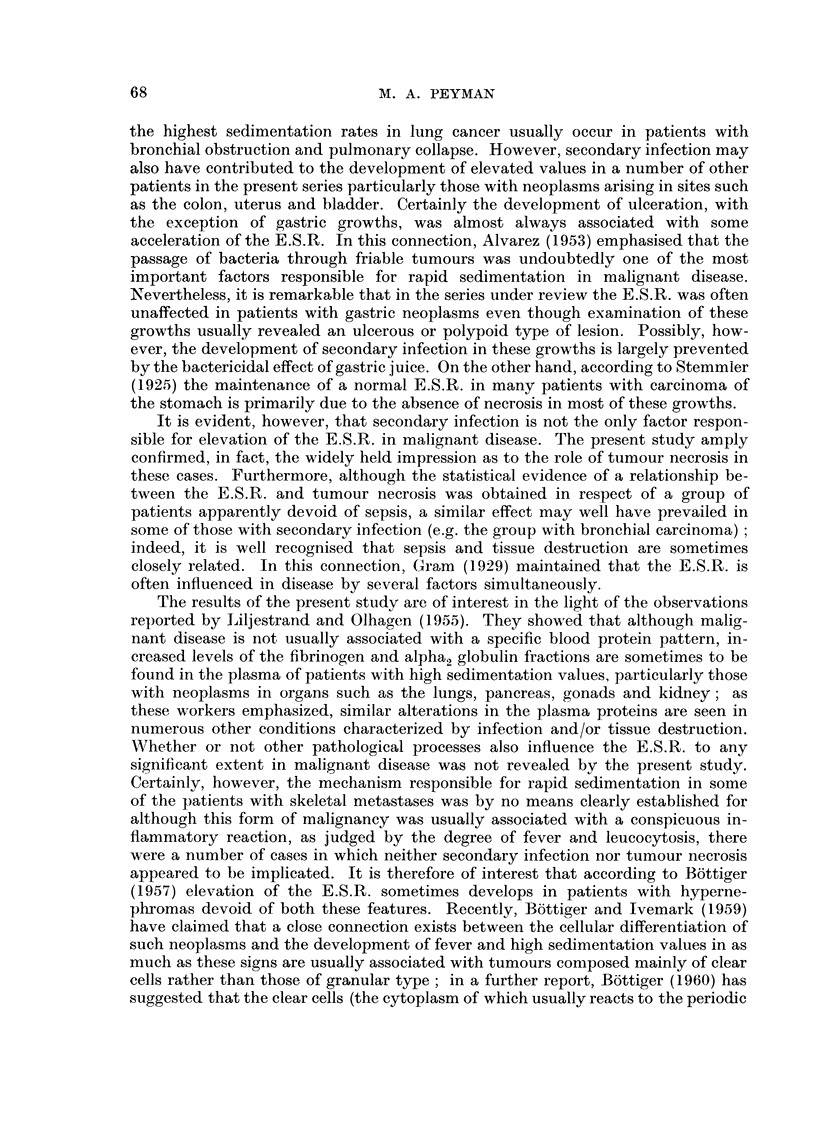

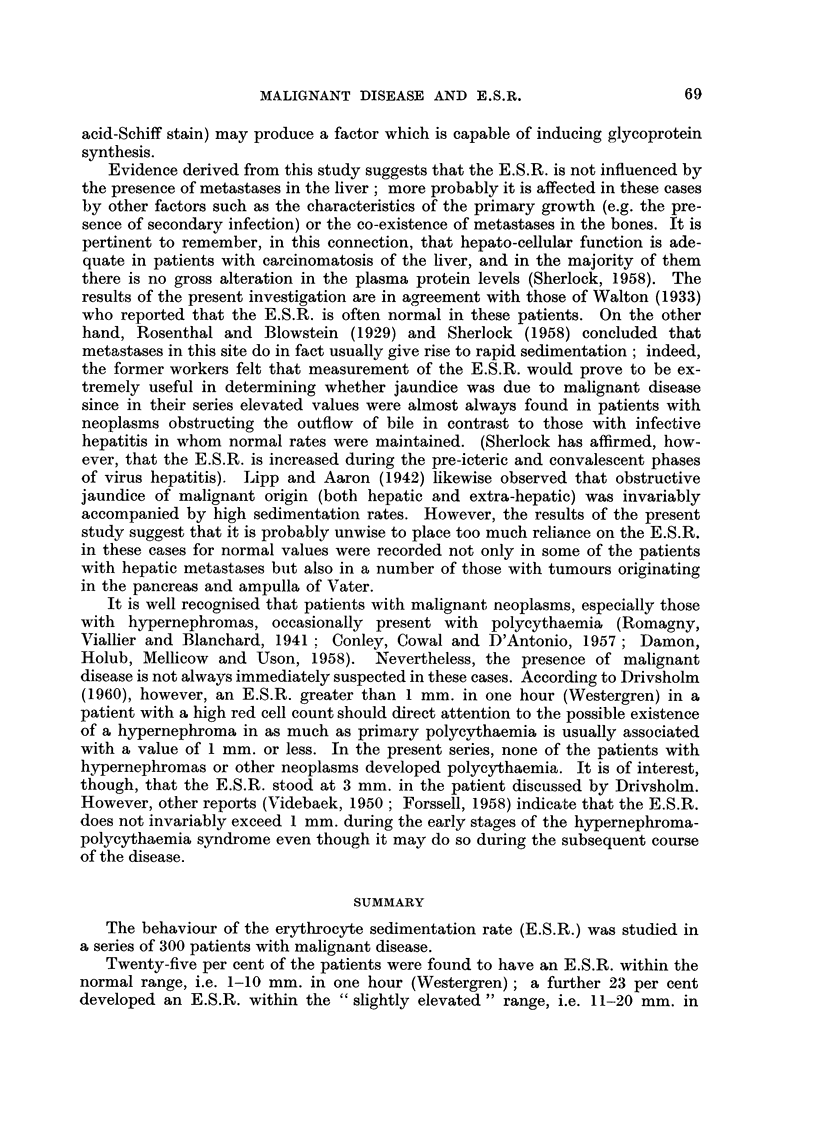

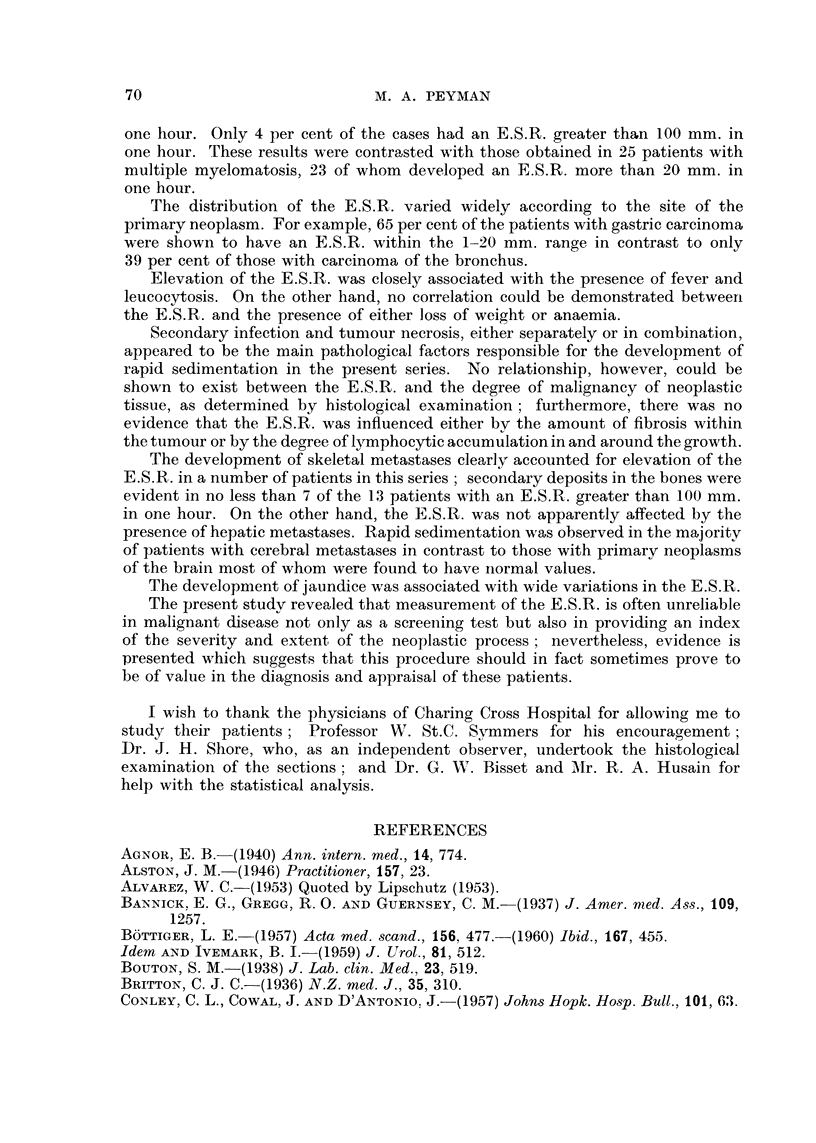

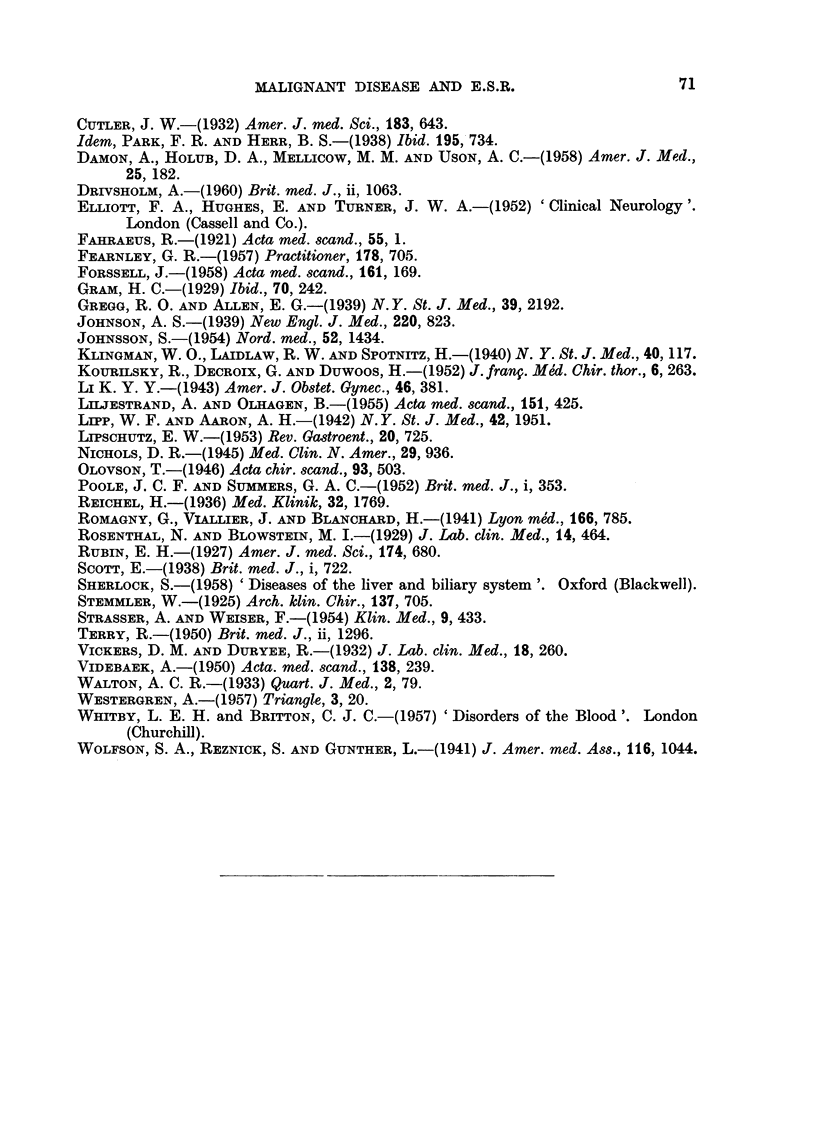


## References

[OCR_01019] BOTTIGER L. E., IVEMARK B. I. (1959). The structure of renal carcinoma correlated to its clinical behavior.. J Urol.

[OCR_01033] DAMON A., HOLUB D. A., MELICOW M. M., USON A. C. (1958). Polycythemia and renal carcinoma.. Am J Med.

[OCR_01037] DRIVSHOLM A. (1960). Hypernephroma and polycythaemia.. Br Med J.

[OCR_01046] FORSSELL J. (1958). Nephrogenous polycythaemia.. Acta Med Scand.

[OCR_01050] JOHNSSON S. (1954). Feber vid nephrom.. Nord Med.

[OCR_01054] KOURILSKY R., DECROIX G., DUWOOS H. (1952). La vitesse de sédimentation des hématies dans les cancers broncho-pulmonaires.. J Fr Med Chir Thorac.

[OCR_01057] LILJESTRAND A., OLHAGEN B. (1955). I. Persistently high erythrocyte sedimentation rate; diagnostic and prognostic aspects.. Acta Med Scand.

[OCR_01064] POOLE J. C., SUMMERS G. A. (1952). Correction of E.S.R. in anaemia; experimental study based on interchange of cells and plasma between normal and anaemic subjects.. Br Med J.

[OCR_01074] STRASSER A., WEISER F. (1954). Blutkörperchensenkungsgeschwindigkeit und Karzinomdiagnose.. Klin Med Osterr Z Wiss Prakt Med.

[OCR_01078] VIDEBAEK A. (1950). Polycythaemia vera; coexisting with malignant tumours (particularly hypernephroma).. Acta Med Scand.

